# Nocturnal hypoxemia, blood pressure, vascular status and chronic mountain sickness in the highest city in the world

**DOI:** 10.1080/07853890.2022.2091791

**Published:** 2022-07-04

**Authors:** Elisa Perger, Sébastien Baillieul, François Esteve, Aurélien Pichon, Gzregorz Bilo, Davide Soranna, Stéphane Doutreleau, Yann Savina, Mathilde Ulliel-Roche, Julien V. Brugniaux, Emeric Stauffer, Laura Oberholzer, Connor Howe, Ivan Hannco, Carolina Lombardi, Renaud Tamisier, Jean-Louis Pepin, Samuel Verges, Gianfranco Parati

**Affiliations:** a Istituto Auxologico Italiano, IRCCS, Sleep Disorders Center & Department of Cardiovascular, Neural and Metabolic Sciences, San Luca Hospital, Milan, Italy; b Biostatistic Unit, University of Milano-Bicocca, Milan, Italy; c Univ. Grenoble Alpes, HP2 Laboratory, INSERM U1300, CHU Grenoble Alpes, Grenoble, France; d Inserm UA7, Rayonnement Synchrotron pour la Recherche Biomédicale, Grenoble, France; e Faculty of Sport Sciences, Université de Poitiers, Laboratory Mobility, aging & exercise (MOVE, EA6314), Poitiers, France; f Istituto Auxologico Italiano, IRCCS, Biostatistics unit, Milan, Italy; g Interuniversity Laboratory of Human Movement Biology (LIBM, EA7424), “Red Blood cell and Vascular Biology” team, Univ Lyon – University Claude Bernard Lyon 1, Villeurbanne, France; h Department of Physical Performance, Norwegian School of Sport Sciences, Oslo, Norway; i Centre for Heart, Lung, and Vascular Health, University of British Columbia, Kelowna, Canada

**Keywords:** Hypoxia, high-altitude, chronic mountain sickness, blood pressure variability, sleep apnoea, sleep disordered breathing

## Abstract

**Introduction:**

Chronic mountain sickness (CMS) is a condition characterized by excessive erythrocytosis in response to chronic hypobaric hypoxia. CMS frequently triggers cardiorespiratory diseases such as pulmonary hypertension and right or left heart failure. Ambient hypoxia might be further amplified night-time by intermittent hypoxia related to sleep-disordered breathing (SDB) so that sleep disturbance may be an important feature of CMS. Our aim was to characterize in a cross-sectional study nocturnal hypoxaemia, SDB, blood pressure (BP), arterial stiffness and carotid intima-media thickness (CIMT) in highlanders living at extreme altitude.

**Methods:**

Men aged 18 to 55 years were prospectively recruited. Home sleep apnoea test, questionnaires (short-form health survey; Montreal cognitive assessment; Pittsburgh Sleep Questionnaire Index and the Insomnia severity index), 24-h ambulatory BP monitoring, CIMT and arterial stiffness were evaluated in 3 groups: i) Andean lowlanders (sea-level); ii) highlanders living at 3,800 m and iii) highlanders living at 5,100 m. Analyses were conducted in sub-groups according to 1) CMS severity 2) healthy subjects living at the three different altitude.

**Results:**

Ninety-two males were evaluated at their living altitudes. Among the 54 highlanders living at 5,100 m, subjects with CMS showed lower mean nocturnal oxygen saturation (SpO_2_), SpO_2_ nadir, lower pulse wave velocity and higher nocturnal BP variability than those with no-CMS. Lower nocturnal SpO_2_ nadir was associated with higher CMS severity (ß= −0.14, *p*=.009). Among the 55 healthy subjects, healthy highlanders at 5,100 m were characterized by lower scores on quality of life and sleep quality scales and lower mean SpO_2_ compared to lowlanders.

**Conclusions:**

Lower nocturnal SpO_2_ and higher nocturnal BP variability are associated with CMS severity in individuals living permanently at high altitude. The role of lower SpO_2_ and higher nocturnal BP variability in the cardiovascular progression of CMS and in the overall prognosis of the disease need to be evaluated in further studies.

## Introduction

In response to chronic hypobaric hypoxia, permanent high-altitude residents develop physiological adaptations. Despite these adaptations, chronic mountain sickness (CMS) can be observed in 5%–33% of highlanders [1,2]. CMS is a syndrome defined by excessive erythrocytosis (EE; haemoglobin concentration, [Hb] ≥ 21 g dL^−1^ for males), associated with signs and symptoms such as breathlessness, palpitations, dizziness, sleep disturbance, cyanosis, peripheral vein dilatation, headache and tinnitus [2]. CMS is often associated with cardiorespiratory diseases such as pulmonary hypertension, right or left heart failure and systemic arterial dysfunction [3]. Although genetic and systemic pathophysiological alterations have been proposed as potential causes of respiratory, cardiovascular, and hormonal responses to chronic hypoxaemia [4–6], the exact mechanisms underlying EE and CMS are still not completely understood.

Substantial nocturnal hypoxaemia has been reported in highlanders [7–11] chronically exposed to hypobaric hypoxia. Sleep disordered breathing (SDB) is frequently observed in lowlanders ascending to high-altitude [12,13] and is even more prevalent in Peruvian highlanders than in lowlanders [14]. Although discordant results have been reported regarding the difference in sleep apnoea incidence between healthy and CMS highlanders [7,9,10,15], more severe exposure to intermittent hypoxia at night on top of the daily hypoxic environment has been shown to contribute to EE in highlanders with CMS [9,16]. The severity, frequency, and duration of these sleep related intermittent desaturations superimposed to chronic hypoxaemia might lead to EE also through a more pronounced chemoreceptor stimulation [17], associated with an abnormally high sympathetic tone, and other hypoxia-related pathways.

Increased night-time blood pressure (BP) has been specifically reported after acute exposure to high-altitude [18]. Although a direct linear association between altitude and BP has been suggested under prolonged altitude exposure [19,20], data are still limited. The mechanisms involved in the increased BP after hypoxic exposure include an increase in sympathetically-mediated vascular modulation, impaired endothelial function and an increase in blood viscosity due to haemoconcentration [21–23]. However, it is not clear whether this depends on EE per se, on the consequent clinical syndrome and the endothelial impairment, or on the degree of hypoxaemia [16,24,25]. Specifically, no data are available regarding SDB, BP and vascular responses in the setting of chronic hypobaric hypoxia at extreme altitude.

To better elucidate the mechanisms underlying CMS, we jointly evaluated cardiovascular, haematological and nocturnal respiratory parameters in individuals living at La Rinconada (Peru), the highest city in the world [23,26]. The main outcome of this study was to assess SDB, ambulatory BP, arterial stiffness, and carotid intima-media thickness (CIMT) in highlanders living at 5,100 m, as a function of their CMS status. Additionally, we described the same parameters among healthy lowlanders (80 m), healthy (i.e. without CMS) highlanders living at 3,800 m and at 5,100 m, to assess the possible adaptation mechanisms associated with chronic altitude exposure.

## Methods

### Study design, settings and participants

We performed a cross-sectional study in February 2019, which was part of a larger research programme (*Expedition 5300*) investigating the pathophysiological consequences of living permanently at high-altitude [26]. Men aged 18 to 55 years were prospectively recruited on a voluntary basis in 3 cities at different altitudes in Peru. Participants with a medical history of diabetes, kidney, respiratory and/or cardiovascular diseases were excluded. Participants were considered as lowlanders if they were born at <1,000 m of altitude and were permanently living at sea level (Lima, Peru, 80 m – Lowlanders) and as highlanders if they were born ≥3,800 m and permanently living at 3,800 m (Puno, Peru – Highlanders 3,800 m) or at 5,100–5,300 m (La Rinconada, Peru – Highlanders 5,100 m). Mostly men live in La Rinconada as they work in gold mine facilities [26]. None of the highlanders reported a prolonged stay (more than 5 days) below 3,500 m over the last 3 months before study data collection.

CMS status was assessed using the Qinghai CMS score, a reference clinical questionnaire which assesses: symptoms of breathlessness and/or palpitations, sleep disturbances, cyanosis, paraesthesia, headache, tinnitus, dilatation of veins, and the presence of excessive erythrocytosis ([Hb] ≥ 21 g dL^−1^ for males) [2]. [Hb] was measured *in situ* from a capillary (fingertip) blood sample (HemoCue®Hb201+, HemoCue AB, Ängelholm, Sweden). Based on their score, highlanders were divided into 3 groups: no-CMS (CMS score ≤5), mild CMS (CMS score 6-10), moderate-severe CMS (CMS score ≥11).

Each participant underwent a clinical evaluation and filled in questionnaires in their validated Spanish versions (see supplementary materials for details) assessing: quality of life – short-form health survey (SF-36); cognitive performances – Montreal cognitive assessment (MOCA); subjective sleep quality – Pittsburgh Sleep Questionnaire Index (PSQI) and the Insomnia severity index (ISI). They also underwent tonometry for pulse wave velocity (PWV) measurement, assessments of CIMT by ultrasonography, home sleep apnoea test (HSAT) and a 24-hour ambulatory BP monitoring (ABPM). HSAT and ABPM were not performed during the same night. HSAT and ABPM scoring were done by a trained physician blinded to altitude of residence and CMS status.

The experimental protocol was approved by the ethics committee of the Universidad Nacional Mayor de San Marcos in Lima (CIEI-2019-002). All subjects gave their written informed consent.

### Home sleep apnoea test

HSAT was performed using a validated portable monitoring system (Vista O2; Novacor, Rueil Malmaison, France) recording airflow by nasal cannulas, respiratory effort using impedance signals derived from electrocardiogram electrodes, body position and pulse oxygen saturation (SpO_2_) *via* an oximeter (Nonin Avant® 4100 – Nonin Medical,Plymouth, MN, USA).

Respiratory events were manually scored according to current American Academy of Sleep Medicine (AASM) scoring rules. Only recordings with at least 4 h of valid signals were kept for the analysis.

### Ambulatory blood pressure monitoring

Twenty-four-hour ABPM was performed using a validated oscillometric device (TM2430; A&D Medical, Japan) (see supplementary materials for details). The following variables were computed: 24-h, daytime and night-time average BP and heart rate (HR) values, dipping status, systolic (S)BP and diastolic (D)BP variability calculated as the standard deviation (SD) of the average day, and night values.

### Arterial stiffness

PWV was evaluated between the carotid and femoral artery with individuals lying in the supine position with the SphygmoCor software database (Atcor Medical, Sydney Australia) (see supplementary materials for details).

### Carotid intima-media thickness

High-resolution B-mode ultrasound (Terason µSmart 3200t, Teratech, United States) combined with a 10-MHz multi-frequency linear array probe (15L4 Smart Mark, Teratech, United States) was used to determine CIMT of the common carotid arteries (see supplementary materials for details).

### Statistical analyses

Continuous data are summarized as mean ± SD for variables with normal distribution (evaluated with Shapiro-Wilk’s test) and as median and interquartile range [IQ] in case of non-normal distribution. Categorical variables are shown as numbers and percentages. Anova modelling (or Kruskal-Wallis) was performed to compare clinical continuous variables among highlanders at 5,100 m with no, mild and moderate-severe CMS as well as among lowlanders and healthy highlanders at 3,800 m and at 5,100 m. When the analysis of variance revealed a significant difference (*p*<.05), post-hoc head-to-head comparisons were conducted using Bonferroni method to consider the type I error (alpha). Chi-square (or Fisher test) was used to compare categorical variables. A multivariate linear regression analysis was performed with CMS severity at 5,100 m as dependent variable and age, time of residence at 5,100 m, mean SpO_2_ during the night, nocturnal SpO_2_ nadir, ODI, PWV as independent variables. The latter variables were defined a priori based on their clinical significance and the model was tested for linearity, normality, collinearity and homoscedasticity. SpO_2_ during the night and ODI were subsequently not included in the model as highly correlated with SpO2 nadir. A p value <.05 was considered statistically significant. Statistical analyses were performed using SPSS 19.00 (IBM, Armonk, NY), R software v4.0.3 and RStudio v1.4 (R Core Team (2020), Vienna, Austria).

## Results

Ninety-two males were evaluated at their living altitudes.

### Data from highlanders at 5,100 m according to CMS severity

A total of 54 highlanders at 5,100 m were evaluated. Subjects’ general characteristics, HSAT and ABPM, among highlanders at 5,100 m with no, mild or moderate-severe CMS are shown in Table 1. No difference was found among CMS groups in SF36 physical, SF36 mental, MOCA, PSQI and ISI questionnaires (Table 2).

**Table 1.** General characteristics, home sleep apnoea test and ambulatory blood pressure monitoring among highlanders at 5,100 m according to CMS severity.

	No CMS (*n* = 17)	Mild CMS (*n* = 14)	Moderate-severe CMS (*n* = 23)
Age, yrs	41 [35–49]	44 [35–49]	44 [40–49]
Body mass index, Kg·m^−2^	25 [24–26]	26 [24–28]	26 [24–28]
Smoking (yes)	2 (11.8)	4 (28.6)	9 (39.1)
Haemoglobin, g·dl^−1^	22 [20–24]	23 [22–23]	24 [23–25]*^,#^
Sleep apnoea test			
SpO_2_ daytime, %	86 [82–88]	85 [80–87]	80 [78–84]*^,#^
AHI, n	7 [2–22]	8 [4–11]	7 [0–11]
AHI OSA, n	4 [2–9]	4 [2–12]	6 [3–13]
AHI CSA, n	3 [1–8]	2 [1–7]	3 [1–5]
ODI, n	18 [13–28]	33 [17–38]	43 [21–58]
SpO_2_ night-time, %	79 [77–82]	77 [73–81]	74 [68–80]*
SpO_2_ Nadir, %	67 [58–75]	55 [48–63]	49 [43–63]*
ABPM			
SBP 24 h, mmHg	127 [122–130]	119 [109–127]	126 [122–134]
DBP 24 h, mmHg	77 [71–80]	73 [69–77]	78 [75–81]
SBP Daytime, mmHg	133 [125–139]	128 [122–139]	134 [131–144]
DBP Daytime, mmHg	81 [75–84]	79 [75–83]	82 [79–87]
SBP Night-time, mmHg	110 [99–117]	105 [100–111]	111 [100–116]
DBP Night-time, mmHg	68 [60–72]	65 [57–69]	70 [60–73]
SBP-SD Daytime, mmHg	25.3 [22.3–28.7]	21.4 [10.1–29.0]	26.3 [23.1–30.6]
DBP-SD Daytime, mmHg	20.5 [17.3–21.8]	20.0 [14.3–22.5]	20.4 [17.5–23.9]
SBP-SD Night-time, mmHg	11.0 [9.2–13.2]	11.8 [8.6–14.4]	17.8 [15.4–20.5]*^,#^
DBP-SD Night-time, mmHg	8.5 [7.4–10.6]	9.1 [8.2–10.4]	13.7 [9.3–18.6]*^,#^
Hypertension, n	4 (24)	2 (14)	6 (26)
Non dipper, n	2 (12)	2 (14)	2 (9)
Pulse wave velocity, m·s^−1^	6 [5–8]	7 [6-7]	5 [5–6]
CIMT, mm	0.38 [0.34–0.48]	0.46 [0.35–0.63]	0.50 [0.35–0.63]

Data are expressed as n (%) or as median [interquartile range]. Smoking define the number of subjects with present or past smoking habits.

Abbreviations: CMS: chronic mountain sickness; AHI: apnoea-hypopnea index; OSA: obstructive sleep apnoea; CSA: central sleep apnoea; ODI: oxygen desaturation index; SpO_2_: pulse oxygen saturation; ABPM: ambulatory blood pressure monitoring; SBP: systolic blood pressure; DBP: diastolic blood pressure; SD: blood pressure variability; CIMT: carotid intima-media thickness.

*Significantly different from no CMS; ^#^Significantly different from mild CMS.

P values <.05 were retained as statistically significant.

**Table 2.** Questionnaire results among highlanders at 5,100 m of altitude according to CMS severity.

	No CMS (*n* = 17)	Mild CMS (*n* = 14)	Moderate-severe CMS (*n* = 23)
SF36 physical	55 [36–71]	55 [46–71]	51 [35–58]
SF36 mental	63 [50–79]	66 [53–76]	59 [40–60]
MOCA	21 [11–22]	20 [17–25]	21 [19–23]
PSQI	8 [5–9]	6 [5–8]	7 [5–9]
ISI	7 [4–10]	5 [2–11]	10 [6–12]

Data are expressed as n (%) or as median [interquartile range].

Abbreviations: CMS: chronic mountain sickness; SF36 physical: short-form health survey to evaluate physical function; SF36 mental: short-form health survey to evaluate mental status; MOCA: Montreal cognitive assessment; PSQI: Pittsburgh Sleep Questionnaire Index; ISI: Insomnia severity index. P values <.05 were retained as statistically significant.

**Figure 1.**
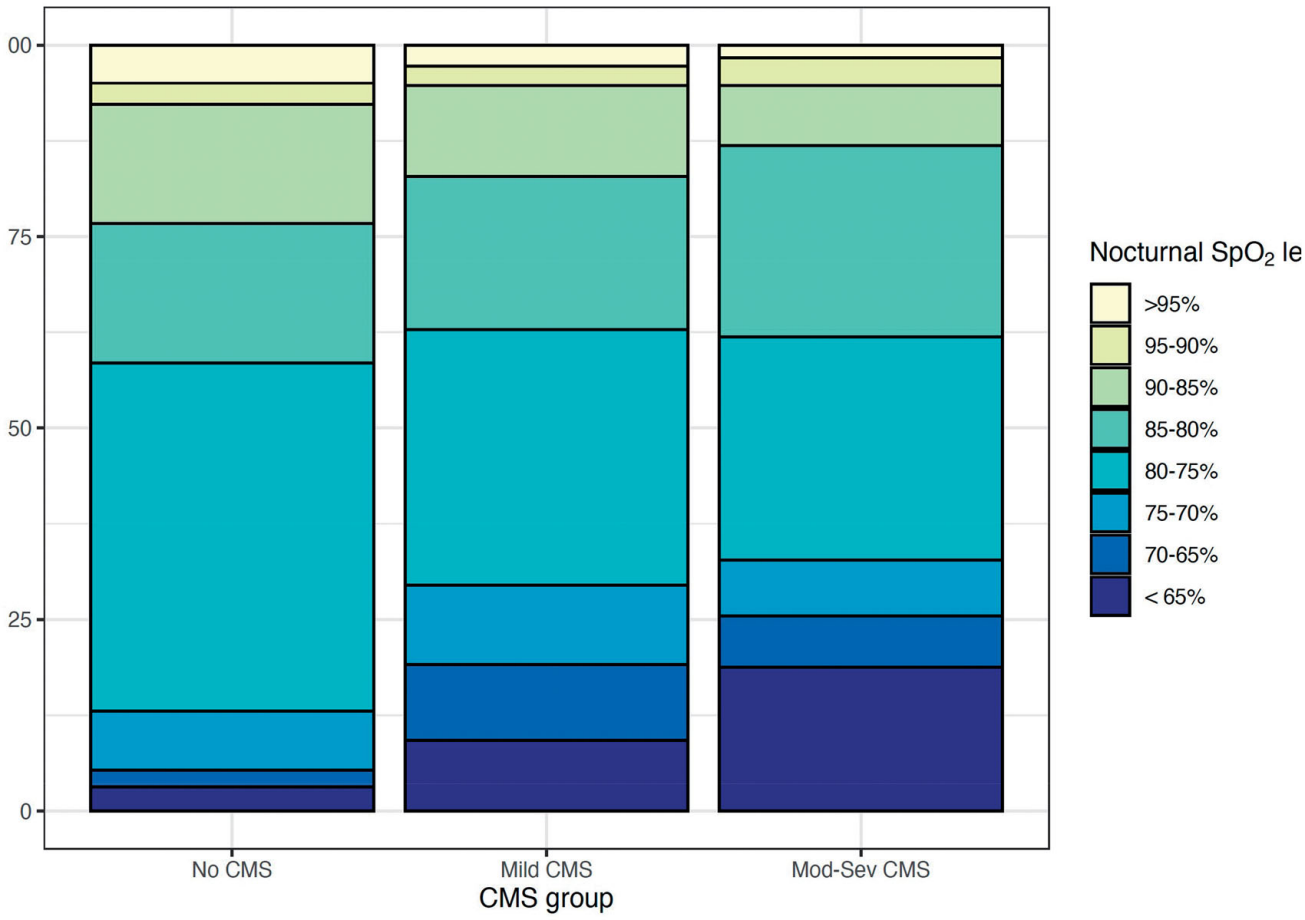
Stratification of nocturnal SpO_2_ in Highlanders at 5,100 m according to CMS status. Nocturnal SpO_2_ levels are calculated from nocturnal pulse oximetry recordings and represent percentage of recording time spent at a specific SpO_2_ value. The percentage of total recording time with a SpO_2_ <65% was different between no CMS and moderate-severe CMS groups (*p*=.02). Abbreviations: Perc., percentage; SpO_2_, pulsed oxygen saturation in dioxygen; CMS, chronic mountain sickness.

*HSAT*: Highlanders at 5,100 m with no CMS compared to highlanders at 5,100 m with mild and moderate-severe CMS showed higher daytime SpO_2_, mean and nadir nocturnal SpO_2_ (see Table 1 and Figure 1 for differences in SpO_2_ distribution among groups). The delta between daytime and night-time SpO_2_ was not significantly different among the groups. Highlanders with mild and moderate-severe CMS presented a tendency towards a higher oxygen desaturation index (ODI) compared to no-CMS highlanders (*p*=.054), while no differences were found in AHI.

*ABPM*: Highlanders with moderate-severe CMS showed higher nocturnal BP variability (BP-SD) than those with mild and no-CMS for both SBP-SD (*p* = 0.020) and DBP-SD (*p*=.026; Table 1).

*Arterial stiffness and CIMT*: A slight but not significant difference was found among groups in PWV (*p*=.08). Although CIMT tended to be higher in highlanders with moderate-severe CMS, differences among groups did not reach statistical significance (*p*=.29; Table 1).

*Multivariate analyses*: The only independent determinant of higher CMS severity was the SpO_2_ nadir (ß= −0.12, 95% confidence interval (CI) [−0.23; −0.02], *p* = 0.03).

### Data from healthy individuals without CMS according to altitude of residency

A total of 55 healthy individuals without CMS were evaluated. Subjects’ general characteristics, HSAT and ABPM data of lowlanders, highlanders at 3,800 m and highlanders at 5,100 m are summarized in Table 3. Highlanders at 5,100 m were older than those at 3,800 m and lowlanders. Regarding questionnaires, highlanders at 5100 m presented worse results in both SF-36, MOCA and PSQI than lowlanders and highlanders at 3800 m (Table 4).

**Table 3.** General characteristics, home sleep apnoea test and ambulatory blood pressure monitoring among lowlanders and healthy highlanders at 3,800 and 5,100 m of altitude.

	Lowlanders (*n* = 19)	Highlanders at 3,800 m (*n* = 19)	Highlanders at 5,100 m (*n* = 17)
Age, yrs	28 [24–35]	25 [23–41]	41 [35–49]*^,#^
Body mass index, Kg·m^-2^	24 [22–28]	25 [22–27]	26 [24–26]
Smoking (yes)	1 (5.3)	0 (0.0)	2 (11.8)
Haemoglobin, g·dl^-1^	15 [14–15]	19 [17–19]^§^	22 [20–24]*^,#^
Sleep apnoea test			
SpO_2_ daytime, %	98 [98–98]	93 [91–94]^§^	86 [82–88]*^,#^
AHI, n	3 [2–7]	9 [6–16]	7 [2–22]
AHI OSA, n	4 [2–6]	5 [3–13]	4 [2–9]
AHI CSA, n	1 [0–2]	3 [1–6]	3 [1–8]
ODI, n	3 [1–6]	13 [9–19]	13 [13–28]
SpO_2_ night-time, %	96 [95–96]	84 [82–87]^§^	79 [77–82]*^,#^
SpO_2_ Nadir, %	89 [88–90]	78 [74–81]^§^	67 [58–75]*^,#^
ABPM			
SBP 24 h, mmHg	129 [119–132]	117 [110–126]	127 [122–130]
DBP 24 h, mmHg	70 [67–77]	71 [69–74]	77 [71–80]
SBP Daytime, mmHg	133 [121–140]	124 [118–139]	134 [125–139]
DBP Daytime, mmHg	74 [70–81]	74 [71–79]	81 [75–84]
SBP Night-time, mmHg	117 [109–122]	101 [97–111]	110 [99–116]
DBP Night-time, mmHg	65 [60–69]	60 [57–66]	68 [60–72]
SBP-SD Daytime, mmHg	17.9 [15.9–21.9]	21.2 [12.4–23.3]	25.3 [22.3–28.7]
DBP-SD Daytime, mmHg	15.6 [14.2–19.9]	17.6 [12.6–20.8]	20.5 [17.3–21.8]
SBP-SD Night-time, mmHg	13.1 [10.4–19.5]	8.6 [4.9–13.7]	11.0 [9.2–13.2]
DBP-SD Night-time, mmHg	8.7 [7.5–13.5]	10.5 [5.9–14.3]	8.5 [7.4–10.6]
Hypertension, n	6 (32)	2 (11)	4 (24)
Non dipper, n	5 (26)	3 (16)	2 (12)
Pulse wave velocity, m·s^-1^	6 [5–7]	7 [6–7]	6 [5–8]
CIMT, mm	0.48 [0.00–0.63]	0.42 [0.35–0.53]	0.37 [0.34–0.43]

Data are expressed as n (%) or as median [interquartile range]. Smoking define the number of subjects with present or past smoking habits.

Abbreviations: AHI: apnoea-hypopnea index; OSA: obstructive sleep apnoea; CSA: central sleep apnoea; ODI: oxygen desaturation index; SpO_2_: pulse oxygen saturation; ABPM: ambulatory blood pressure monitoring; SBP: systolic blood pressure; DBP: diastolic blood pressure; SD: blood pressure variability; CIMT: carotid intima-media thickness.

*Highlanders 5,100 m significantly different from Lowlanders; ^#^Highlanders 5,100 m significantly different Highlanders at 3,800 m; ^§^Highlanders 3,800 m significantly different from Lowlanders. P values <.05 were retained as statistically significant.

**Table 4.** Questionnaire results among lowlanders and healthy highlanders at 3,800 and 5,100 m of altitude.

	Lowlanders (*n* = 19)	Highlanders at 3,800 m (*n* = 19)	Highlanders at 5,100 m (*n* = 17)
SF36 physical	86 [59–93]	79 [70–93]	55 [36–71]*^,#^
SF36 mental	79 [63–90]	79 [66–90]	63 [50–79]*^,#^
MOCA	26 [24–29]	27 [25–28]	21 [11–22]*^,#^
PSQI	5 [2–7]	4 [3–6]	8 [5–9]*^,#^
ISI	5 [2–9]	7 [1–10]	7 [4–10]

Data are expressed as n (%) or as median [interquartile range].

Abbreviations: SF36 physical: short-form health survey to evaluate physical function; SF36 mental: short-form health survey to evaluate mental status; MOCA: Montreal cognitive assessment; PSQI: Pittsburgh Sleep Questionnaire Index; ISI: Insomnia severity index.

*Significantly different from Lowlanders; ^#^Significantly different from Highlanders at 3,800 m. P values <.05 were retained as statistically significant.

**Figure 2.**
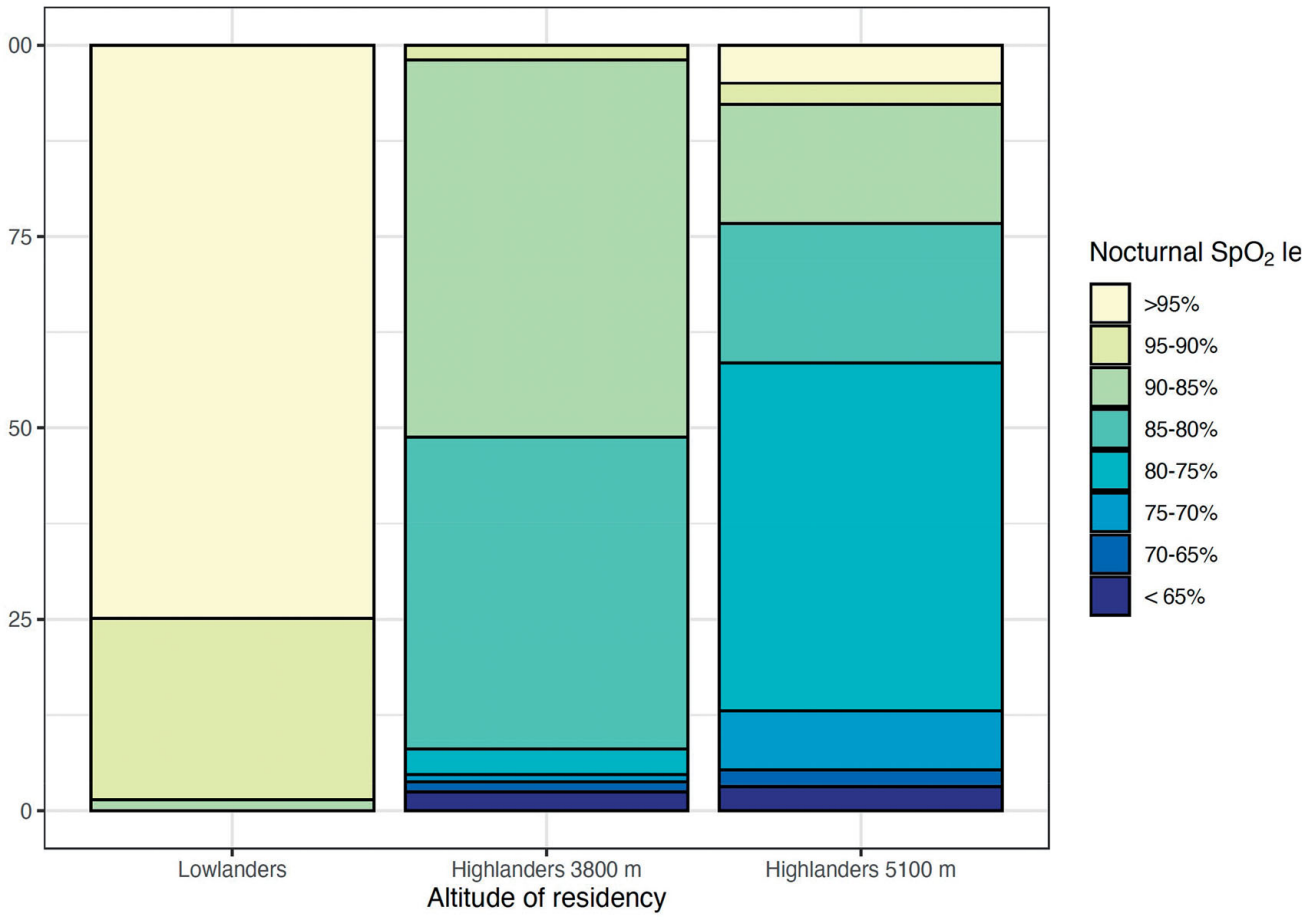
Stratification of nocturnal SpO_2_ in healthy subjects according to the altitude of residency. Nocturnal SpO_2_ levels are calculated from nocturnal pulse oximetry recordings and represent percentage of recording time spent at a given SpO_2_ value. Distribution of SpO_2_ during the night differed significantly among the three groups (all *p*<.01). Abbreviations: Perc.: percentage; SpO_2_: pulsed oxygen saturation.

*HSAT:* Daytime SpO_2_, mean nocturnal SpO_2_ and SpO_2_ nadir decreased from lowlanders to highlanders at 3,800 m and at 5,100 m (*p*<.05 for all the variables). These results were also confirmed when adjusting the model for age and haemoglobin values as shown in the supplemental material. Distribution of SpO_2_ during the night differed significantly among the three groups (*p*<.01; Figure 2): in lowlanders SpO_2_ remained above 95% in most cases, in highlanders at 3,800 m it ranged between 80% and 90% and in highlanders at 5,100 m it dropped to the range 80%–75%.

*ABPM*: Daytime and night-time DBP tended to be higher in highlanders at 5,100 m, but this difference was not statistically significant. No difference among groups emerged in hypertension status, BP dipping and variability of BP (Table 3).

*Arterial stiffness and CIMT:* No differences were observed among groups for PWV (*p*=.72) or CIMT (*p*=.52) (Table 3).

## Discussion

Here, we provide the first ever data on nocturnal oxygenation and SDB, 24-h BP, arterial stiffness and CIMT in residents of the highest city in the world (5,100–5,300 m) according to their CMS status. We also report the differences in these variables among healthy lowlanders and healthy highlanders living at 3800 and 5100 m, respectively. The main results of our study are: i) among highlanders at 5,100 m, individual with moderate-severe CMS presented the lowest daytime and night-time SpO_2_, with increased BP variability during the night and with a tendency towards worse ODI; ii) lower SpO_2_ nadir was independently associated with more severe CMS in highlanders at 5,100 m; iii) among healthy individuals, both daytime and night-time SpO_2_ were reduced in highlanders, these reductions being more pronounced at extreme altitude.

### Questionnaires

Healthy highlanders living at 5,100 m presented higher PSQI scores, reflecting reduced subjective sleep quality. This may have contributed, together with the tough living conditions, to the reduced SF36 physical and mental scores observed in this group. Cognitive performances as assessed by MOCA scores were also significantly impaired in healthy highlanders at 5,100 m compared to healthy lowlanders and healthy highlanders at 3,800 m, as previously reported [27]. Chronic altitude exposure, older age and sleep disturbances through systemic inflammation [28] may represent potential mechanisms underlying those impairments. Altogether, healthy highlanders at 5,100 m presented indicators of impaired quality of life and cognition that may arise from several environmental and lifestyle factors. Intriguingly, CMS presence and severity did not seem to worsen any of these parameters. By contrast, previous reports [28] have suggested that CMS is associated with cognitive decline and depression. However, it is noteworthy that this study was conducted at a much lower altitude (3,600 m). Taken together, at the extreme altitude of La Rinconada (5,100 m), chronic hypoxic exposure and tough living conditions might contribute to the impaired sleep quality and quality of life observed in these high-altitude dwellers.

### Sleep-disordered breathing and nocturnal hypoxaemia

We demonstrated severe nocturnal hypoxaemia among highlanders at 5,100 m either with or without CMS, with no evidence of increased sleep apnoea prevalence, in accordance with previous studies [9,11], while others reported higher AHI in highlanders with EE [7,10,15]. Despite this discrepancy in the literature, all studies stated that highlanders with CMS are characterized by lower daytime and night-time SpO_2_ than their healthy counterparts [8–11,17]. Accordingly, we found a similar nocturnal drop in SpO2 from daytime values among CMS groups without differences in apnoea severity. Thus, it is possible that the lower nocturnal SpO2 we observed might be a direct consequence of the worse daytime hypoxaemia in CMS subjects. Spicuzza et al. [9] showed that at 4,380 m, mean night SpO_2_ in highlanders with EE was 83.7%, with 38% of the time spent between 76%–80% versus a mean SpO_2_ of 85.6% and the majority of the time spent over 81% in healthy highlanders. Our results further highlight that an increase in the altitude of residency worsens nocturnal hypoxaemia and that the more severe the CMS the lower the mean nocturnal SpO_2_ and SpO_2_ nadir. While it has been recently reported that high erythropoietin (EPO) level is associated with low mean nocturnal SpO_2_, links between EPO metabolism and SpO_2_ nadir have not been evaluated [29]. Nocturnal oxygen saturation drops in susceptible highlanders characterized by low arterial oxygen pressure (PaO_2_) may be explained by being in the steepest part of the SpO_2_/PaO_2_ curve, where small changes in PaO_2_ due to respiratory instability (not yet reaching the definition of hypopnea/apnoea) can induce significant reductions in SpO_2_. Besides such tonic reduction, this phenomenon might easily determine chronic intermittent drops of SpO_2_, which are known to increase [Hb] in highlanders with CMS [9,16]. The evidence of higher nocturnal BP variability supports such an interpretation and, together with the ODI, suggests a slight contribution of episodes of phasic events rather than the implication of tonic desaturations only. Moreover, despite a lack of statistical significance, ODI tended to be higher among highlanders with moderate-severe CMS (*p*=.054). However, altogether, our results suggest that ODI, and even more so AHI, may not be appropriate to precisely characterize nocturnal breathing of highlanders at 5,100 m. Further studies should focus on more sensible indices to better evaluate nocturnal breathing pattern and hypoxic burden of highlanders and their relationship with CMS.

### Ambulatory blood pressure monitoring

In our study the presence and severity of CMS were not associated with significant differences in ambulatory BP, either during daytime or during night-time. While a number of studies documented BP increase, in particular at night, during acute HA exposure [18], the evidence on systemic BP and arterial hypertension in highlanders is limited [19,20,30]. A meta-regression analysis of studies in Tibetan highlanders indicated a possible trend towards higher BP values with increasing altitude [30]. Only two studies in highlanders used 24-h ABPM as in the present study, an approach characterized by higher accuracy and allowing to assess nocturnal BP [24,31]. The article by Bilo et al. [31] reported a significantly higher prevalence of ambulatory hypertension in subjects with EE. Our results are discordant, possibly due to: 1) our participants being recruited at a higher altitude and even among those without CMS, only a few had [Hb] < 21 g dL^−1^, while in the study of Bilo et al. [31], EE alone and not the presence of CMS (EE plus symptoms) was independently associated with hypertension; 2) the inclusion of miners, i.e. physically active subjects.

Interestingly, in our participants with moderate-severe CMS, we observed a significantly higher nocturnal BP variability. A plausible explanation could be the severe nocturnal drops in SpO_2_ observed in this group. In fact, repeated slight desaturations might cause transient, chemoreflex-mediated increases in sympathetic activity. Such increases might be too short-lasting to significantly alter average BP levels but might increase BP fluctuations during the night. Another possible mechanism is related to the inhibition of the renin-angiotensin-aldosterone system, known to occur during hypoxic exposures to a very high-altitude [18,32]. Dysfunction of these key pathways might reduce the stability of BP homeostasis leading to increased BP variability. Of note, our finding may have clinical relevance, as higher BP variability in previous studies has been associated to increased cardiovascular and all-cause mortality [33,34].

### Arterial stiffness and CIMT

We found no significant difference in PWV and CIMT between altitude and CMS groups. Regarding the effect of altitude, our results are consistent with those of a previous study reporting no difference in PWV between Himalayan dwellers (living permanently at altitude ranging between 2,600 and 3,800 m) compared to Caucasian subjects living at sea-level [35]. The same group reported differences in carotid vascular architecture including a thinner CIMT in native Himalayan dwellers [35], whereas we reported no significant difference in CIMT among altitude groups. Nonetheless, it should be noted that Himalyan and Andean dwellers are known to present different responses to the hypoxic environment, in turn making comparisons between these populations difficult [25,36]. Regarding the impact of CMS, our results are conflicting with those of a previous study, showing significantly increased PWV and CIMT in CMS individuals living permanently at 3,600 m [37]. Differences in altitude of residency, age and use of different cut-offs of CMS score could have contributed to these discrepant results. Altogether, the main determinants of the changes in vascular architecture and function as well as their significance in terms of adaptive or maladaptive processes to permanent high-altitude residence remain to be further elucidated.

## Limitations

Although the exclusion of subjects affected by cardiovascular and respiratory diseases permitted to avoid cofounders for our results, this choice might also have excluded from our analyses those patients with very severe CMS status. The extreme conditions in which we operated required the use of device that cannot guarantee a high-quality signal. Although type II portable device for SDB diagnosis are also used in a hospital setting for clinical evaluations [38], this type of devices might not be able to accurately detect sleep apnoea in certain subject groups [39,40]. Moreover, the sleep data of the present study might be impacted by the absence of bands for thoracic-abdominal motion with the impossibility to differentiate central and obstructive events. Other limitations of our study are the impossibility to evaluate lung function due to technical problems and the absence of a fitness status comparison. Thus, exposure to mining dust could not be ruled out as additional factor contributing to hypobaric hypoxia at the extreme altitude we performed our project.

## Conclusions

In the extreme living conditions in the highest city in the world, low levels of SpO_2_ seem to be a preponderant feature associated with maladaptation (i.e. CMS) of some highlanders to chronic hypobaric hypoxia. CMS subjects are characterized by lower nocturnal SpO_2_ and SpO_2_ nadir, known as major contributors to EE, together with heightened nocturnal BP variability, suggesting sympathetic over-activity. The role of mechanism underlying our findings in the cardiovascular progression of CMS and in the overall prognosis of the disease need to be evaluated in further studies.

## Supplementary Material

Supplemental Material

## Data Availability

The data that support the findings of this study are available from the corresponding author, EP, upon reasonable request.
