# Supplementary material for: Nocturnal hypoxemia, blood pressure, vascular status and chronic mountain sickness in the highest city in the world
Source: Ann Med. 2022 Jul 4;54(1):1884–93. doi: 10.1080/07853890.2022.2091791 (PMC9258438; doi:10.1080/07853890.2022.2091791)
Supplement: Supplemental Material [file IANN_A_2091791_SM5633.docx]

**Supplementary material**

**METHODS**

***Questionnaires***

- Quality of life was assessed using the short-form (36) health survey (SF-36) [1] in its validated Spanish version [2]. The questionnaire comprises 36 items classified in 8 subscales: physical functioning (10 items), role physical (4), bodily pain (2), general health (5), vitality (4), social functioning (2), role emotional (3), and mental health (5). It also provides two summary measures: a physical component summary and a mental health summary.
- Subjective sleep quality and disturbances in the last month were self-reported using the Pittsburgh Sleep Questionnaire Index (PSQI) [3] in its Spanish version [4]. The PSQI consists of 19 self-rated questions that generate seven component scores (with subscales ranged 0–3): sleep quality, sleep latency, sleep duration, habitual sleep efficiency, sleep disturbance, use of sleeping medication, and daytime dysfunction. The sum of these seven component scores yields one global score of subjective sleep quality (ranged 0–21), with higher scores representing poorer subjective sleep quality. The five additional questions to be answered by bedmates or roommates, used only for clinical information, were not analyzed.
- The Spanish [5] version of the Insomnia severity index (ISI) [6]. The ISI is a 7-item self-report questionnaire assessing the nature, severity and impact of insomnia over the last month. The dimensions assessed are the severity of sleep onset, sleep maintenance, early awakenings problems, sleep dissatisfaction, interference of sleep difficulties with daytime functioning, noticeability of sleep problems by others, and distress caused by the sleep difficulties. A 5-point Likert scale is used to rate each item and total score range from 0 to 28. The total score is interpreted as follows: absence of insomnia (0-7); sub-threshold insomnia (8-14); moderate insomnia (15-21); and severe insomnia (22-28).
- Montreal cognitive assessment (MOCA) [7] is a 30-point test, validated in Spanish and used in Latin America [8, 9]. This test evaluates overall neurocognitive function through the assessment of eight cognitive domains. A score >25/30 is considered as normal, scores ranging between 18-25 reflects a mild, 10-17 a moderate and <10 a severe neurocognitive impairment (<https://www.mocatest.org/faq/>).

***Home Sleep Apnoea Test***

HSAT was performed over a single night using a validated portable monitoring system (Vista O2; Novacor, Rueil Malmaison, France). The following physiological variables were recorded [10]: airflow by nasal cannulas, respiratory effort using impedance signals derived from electrocardiogram electrodes, body position and pulse oxygen saturation (SpO_2_) by an oximeter (Nonin Avant^®^ 4100 - Nonin Medical, Plymouth, MN, USA) at a sampling rate of 1 Hz.

Respiratory events were manually scored according to current American Academy of Sleep Medicine (AASM) scoring rules [11, 12]. Oxygen desaturation index (ODI), mean nocturnal SpO_2_ and distribution of recording time spent at specific SpO_2_ levels were also calculated. Only recordings with at least 4 hours of valid signals were kept for the analysis.

***Ambulatory blood pressure monitoring***

Twenty-four-hour ABPM was performed using a validated oscillometric device (TM2430; A&D Medical, Japan) applied on the non-dominant arm. The measurements were scheduled every 15 minutes during daytime (6.00–23.00 h) and every 20 minutes during night-time (23.00–6.00 h). Patients were asked to stay still during each measurement and to keep a standardized activity diary. Self-reported awake and asleep periods, based on the logbook entries, were applied in the analyses.

ABPM recordings were considered as valid if at least 70% of expected readings were available and if recordings did not contain two or more consecutive hours without valid readings. The following variables were computed: 24-h, daytime and night-time average BP and heart rate (HR) values, dipping status, systolic (S)BP and diastolic (D)BP variability calculated as the standard deviation (SD) of the average day, and night values. Dipping status was defined as a nocturnal BP fall lower than 10% of the daytime average BP value.

***Arterial stiffness***

PWV was evaluated between the carotid and femoral artery with individuals lying in the supine position [13]. The distance from carotid to femoral artery was measured with a steel tape measure, and determined by subtracting the suprasternal-notch to carotid site distance from the suprasternal-notch to femoral site distance. Pulse measurements were performed using the SphygmoCor probe over the carotid and femoral artery, while an ECG recording was performed simultaneously. The values were entered into the SphygmoCor software database (Atcor Medical, Sydney Australia). Carotid-femoral PWV was measured by dividing the distance between the sampling sites by the time difference between the respective delays in the onset of femoral and carotid pulses with regard to the preceding R wave of an electrocardiographic recording. A minimum of 12 s of signal (approximately 10 heart beats) was recorded after a strong accurate and reproducible pulse wave signal was obtained.

***Carotid intima-media thickness***

High-resolution B-mode ultrasound (Terason µSmart 3200t, Teratech, United States) combined with a 10-MHz multifrequency linear array probe (15L4 Smart Mark, Teratech, United States) was used to determine CIMT of the common carotid artery, after at least 30 min of rest in a supine position. With the head in a slightly bent position towards the left to allow scanning of the right common carotid artery (CCA) the ultrasound transducer was applied with an angle of 90° in relation to the vessel walls. Scanning of the CIMT was made in the CCA at least 1 cm from the bulb. Screen captures of the ultrasound was saved as a video file (Camtasia Studio, TechSmith Co, Ltd, United States) for offline analysis. Bins of 30-s video images were analysed using an automated edge-detection and wall-tracking software (Vascular tools 5, Medical Imaging Applications, Iowa, USA), which automatically tracks the walls of the CCA within the defined region of interest. CIMT values were calculated as the average over the duration of the 30-s video.

**RESULTS**

***Data from healthy individuals without CMS according to altitude of residency***

In healthy individuals, both daytime and night-time SpO_2_ were reduced in a more pronounced way at extreme altitude. To exclude the potential confounding role of other variables, a pairwise comparison between least-square means for the three altitudes of residency was calculated with a linear model including age and [Hb]. We adjusted the inflation of type I error with Tukey method. All comparisons were statistically significant as shown in table S1.

| Comparison | LS means difference | P-value adjusted with Tukey |
| --- | --- | --- |
| *SpO_2_ diurnal* |  |  |
| Sea level vs 3800m | 10.93 | <.0001 |
| Sea level vs 5100m | 17.91 | <.0001 |
| 3800m vs 5100m | 6.98 | 0.0008 |
| *SpO_2_ nocturnal* |  |  |
| Sea level vs 3800m | 11.74 | <.0001 |
| Sea level vs 5100m | 16.47 | <.0001 |
| 3800m vs 5100m | 4.73 | 0.0026 |

**SUPPLEMENTAL REFERENCE**

1. Berry RB, Brooks R, Gamaldo CE, Harding SM, Marcus C, Vaughn BV. The AASM manual for the scoring of sleep and associated events. *Rules, Terminology and Technical Specifications, Darien, Illinois, American Academy of Sleep Medicine* 2012: 176: 2012.

2. Alonso J, Prieto L, Anto JM. [The Spanish version of the SF-36 Health Survey (the SF-36 health questionnaire): an instrument for measuring clinical results]. *Med Clin (Barc)* 1995: 104(20): 771-776.

3. Buysse DJ, Reynolds CF, 3rd, Monk TH, Berman SR, Kupfer DJ. The Pittsburgh Sleep Quality Index: a new instrument for psychiatric practice and research. *Psychiatry Res* 1989: 28(2): 193-213.

4. Zhong QY, Gelaye B, Sánchez SE, Williams MA. Psychometric Properties of the Pittsburgh Sleep Quality Index (PSQI) in a Cohort of Peruvian Pregnant Women. *J Clin Sleep Med* 2015: 11(8): 869-877.

5. Fernandez-Mendoza J, Rodriguez-Muñoz A, Vela-Bueno A, Olavarrieta-Bernardino S, Calhoun SL, Bixler EO, Vgontzas AN. The Spanish version of the Insomnia Severity Index: a confirmatory factor analysis. *Sleep Med* 2012: 13(2): 207-210.

6. Morin CM, Belleville G, Belanger L, Ivers H. The Insomnia Severity Index: psychometric indicators to detect insomnia cases and evaluate treatment response. *Sleep* 2011: 34(5): 601-608.

7. Nasreddine ZS, Phillips NA, Bédirian V, Charbonneau S, Whitehead V, Collin I, Cummings JL, Chertkow H. The Montreal Cognitive Assessment, MoCA: a brief screening tool for mild cognitive impairment. *J Am Geriatr Soc* 2005: 53(4): 695-699.

8. Loureiro C, Garcia C, Adana L, Yacelga T, Rodriguez-Lorenzana A, Maruta C. [Use of the Montreal Cognitive Assessment (MoCA) in Latin America: a systematic review]. *Rev Neurol* 2018: 66(12): 397-408.

9. Ojeda N, Del Pino R, Ibarretxe-Bilbao N, Schretlen DJ, Pena J. [Montreal Cognitive Assessment Test: normalization and standardization for Spanish population]. *Rev Neurol* 2016: 63(11): 488-496.

10. Poupard L, Mathieu M, Goldman M, Chouchou F, Roche F. Multi-modal ECG Holter system for sleep-disordered breathing screening: a validation study. *Sleep Breath* 2012: 16(3): 685-693.

11. Medicine AAoS. International classification of sleep disorders. 3rd ed: American Academy of Sleep Medicine, 2014.

12. Kapur VK, Auckley DH, Chowdhuri S, Kuhlmann DC, Mehra R, Ramar K, Harrod CG. Clinical Practice Guideline for Diagnostic Testing for Adult Obstructive Sleep Apnea: An American Academy of Sleep Medicine Clinical Practice Guideline. *J Clin Sleep Med* 2017: 13(3): 479-504.

13. Doupis J, Papanas N, Cohen A, McFarlan L, Horton E. Pulse Wave Analysis by Applanation Tonometry for the Measurement of Arterial Stiffness. *The open cardiovascular medicine journal* 2016: 10: 188-195.
